# Supplementary material for: The Phylogeny and Metabolic Potentials of an Aromatics-Degrading Marivivens Bacterium Isolated from Intertidal Seawater in East China Sea
Source: Microorganisms. 2024 Jun 27;12(7):1308. doi: 10.3390/microorganisms12071308 (PMC11278965; doi:10.3390/microorganisms12071308)

Supplementary Tables and Figures:

Supplementary Tables:

Table S1: The fatty acids contents of strain LCG002.

Table S2: The ANIb values and aligned percentage between strain LCG002 and other strains.

Table S3: The AAI values and aligned percentage between strain LCG002 and other strains.

Table S4: The DDH value and aligned percentage between strain LCG002 and other strains.

Table S5: The glycoside hydrolase genes predicted in strain LCG002.

Table S6: The amino acid related and extracellular (signal-fused) peptidases genes predicted in strain LCG002.

Table S7: The genes involved in utilization of carboxylates.

Table S8: The genes involved in degradation of aromatic monomers and aromatic acids.

Table S9: The genes involved in utilization of carbonmonoxide and hydrogen.

Table S10: The genes involved in utilization of nitrogen, phosphorus and sulfur sources.

Supplementary Figures:

Figure S1: The transmission electron micrograph of strain LCG002.

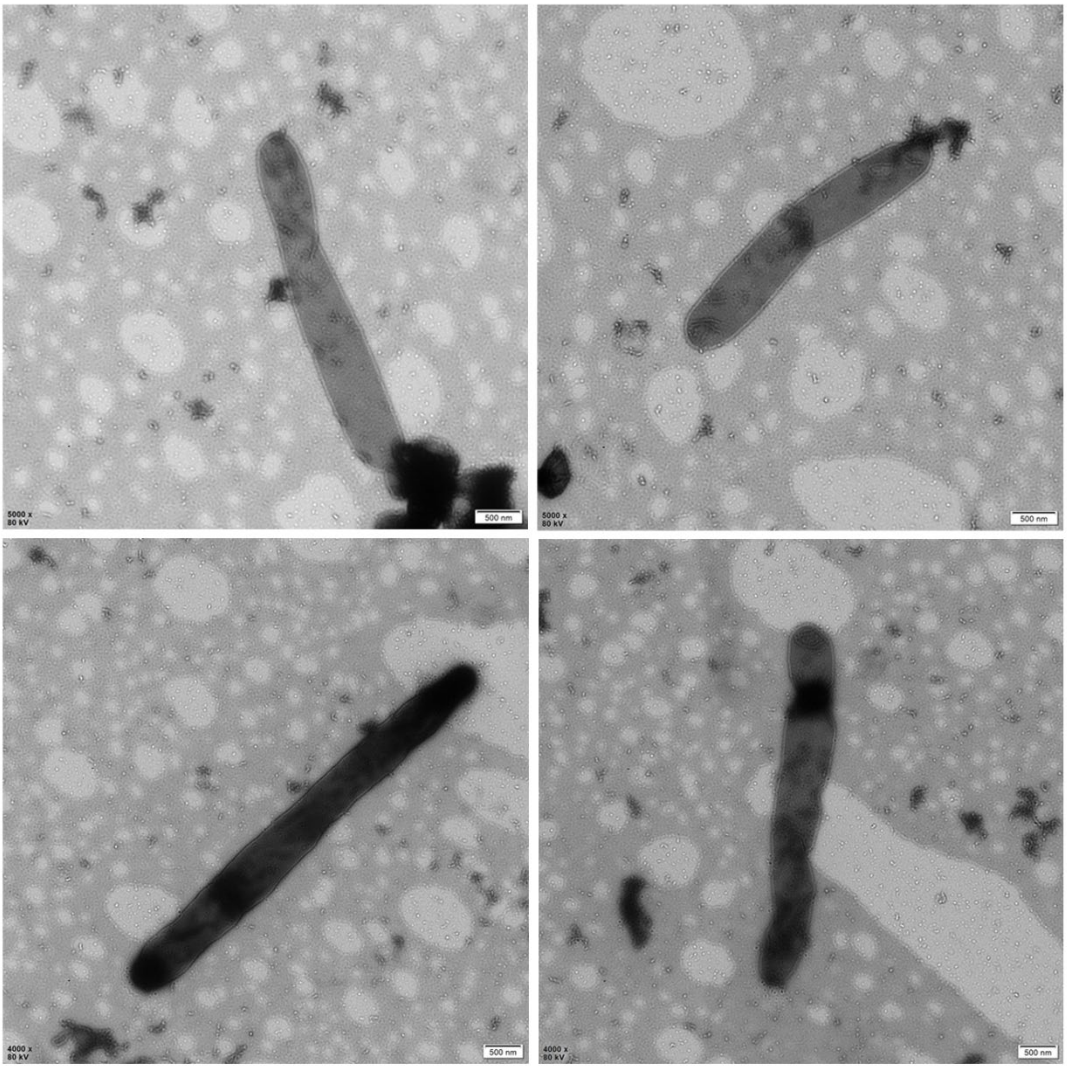

Figure S2: The growth curve of strain LCG002 under multiple temperatures.

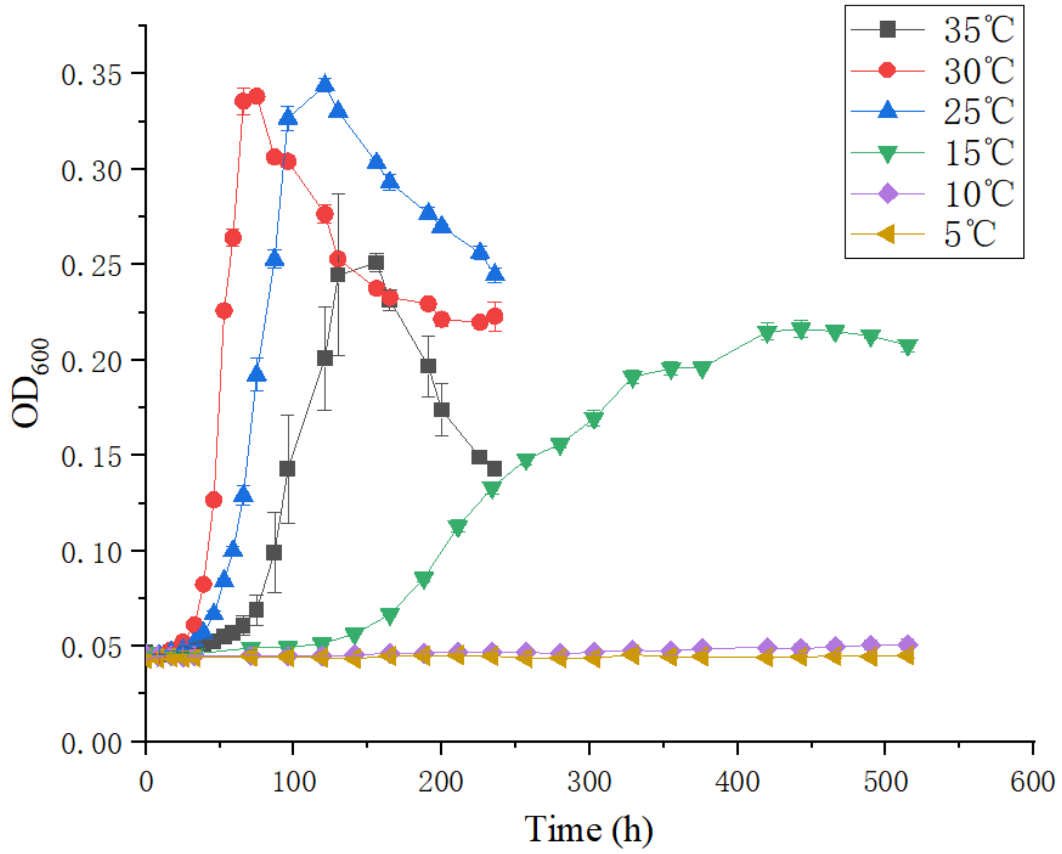

Figure S3: The polar lipids of strain LCG002 were composed of phosphatidylglycerol (PG) and phosphatidylethanolamine (PE), unidentified aminophospholipid (APL), 2 unidentified phospholipid (PL1 and PL2), unidentified aminolipid (AL), 3 unidentified glycolipid (GL1-GL3), 3 unidentified lipid (L1-L3), unidentified phosphatidylglycerol lipid (PGL) and 3 unidentified aminoglycerolipid (AGL1-AGL3).

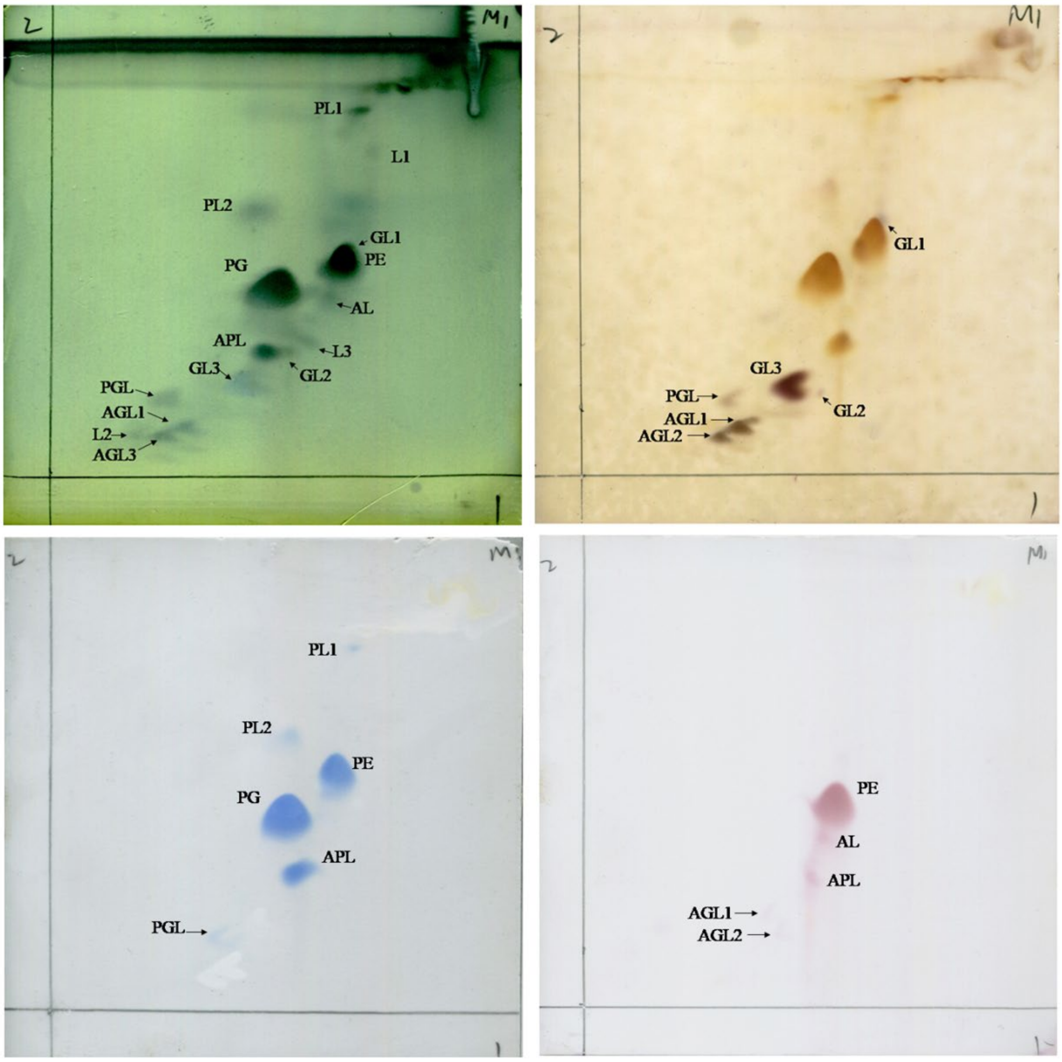

Figure S4: Graphical representation of the *Marivivens* strain LCG002 genomes. The outermost circle is the identification of genome size. The second and third circles are the CDS on the positive and negative strands, and different colors indicate the functional classification of CDS with different COGs. The fourth circle is the rRNAs and tRNAs. The fifth circle is the GC content, and the outward red portion indicates that the GC content of the region is higher than the average GC content of the whole genome, while the inward blue portion indicates that the GC content of the region is lower than the average GC content of the whole genome, and the higher the peak indicates the greater the difference with the average GC content. The innermost circle is the GC-Skew value (G-C/G+C).

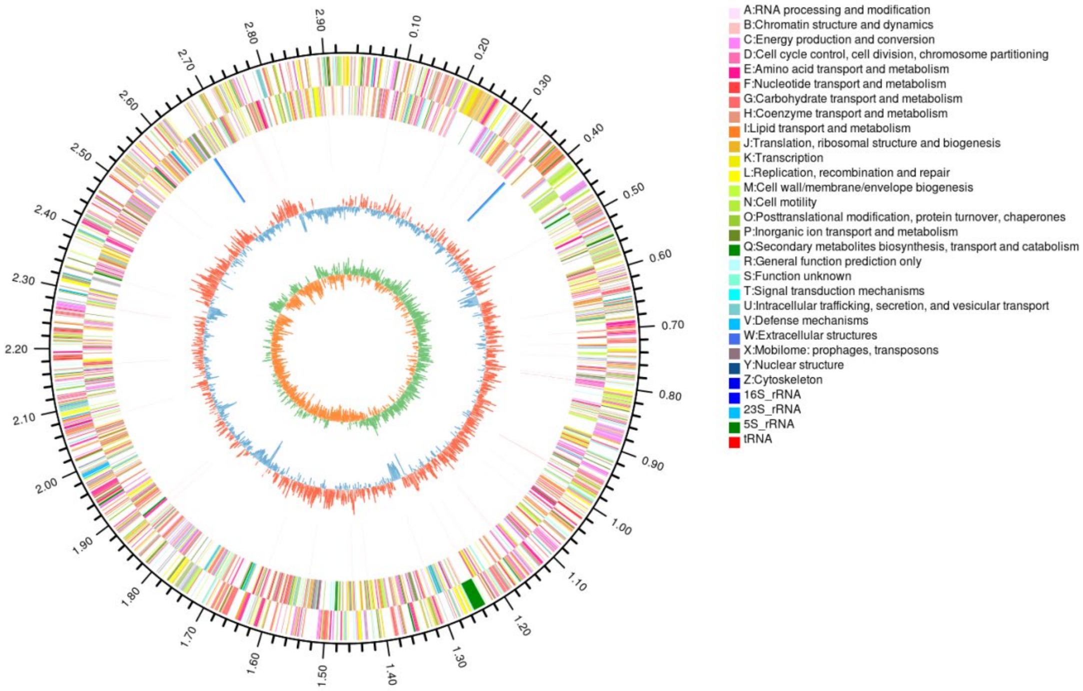

Figure S5: The lignin utilization of strain LCG002. A and B are experimental groups, and C and D are control groups, indicating the counts of live bacteria of strain LCG002 calculated using the SYTO 9/PI live/dead bacteria double strain kit with ASW-lignin medium for 0 and 7 days, respectively. Error bar indicates each sample was with three independent biological replicates. Asterisks indicate t-test significant differences (NS, no significant; *, P value < 0.05; **, P value < 0.01; ***, P value < 0.001; ****, P value < 0.0001).

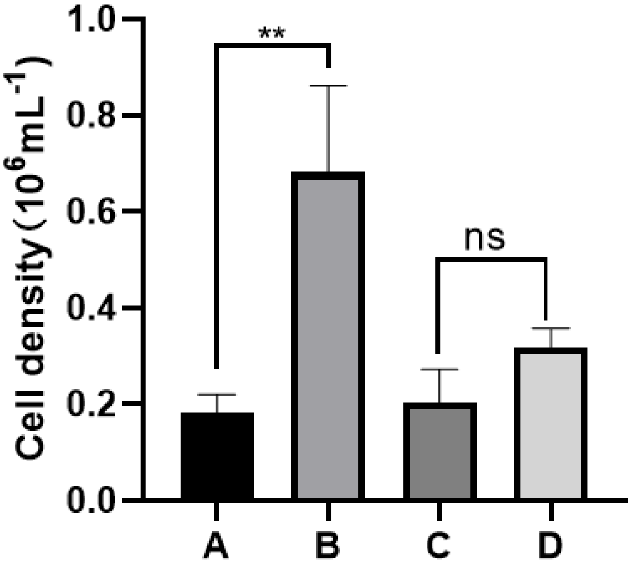

Supplement: Supplementary file 1 [file microorganisms-12-01308-s001.zip › Supplementary Tables and Figures Legends.pdf]
